# The Role of Muscle Biopsy in the Era of Modern Genomic Medicine—A Review

**DOI:** 10.3390/jcm15155906

**Published:** 2026-07-29

**Authors:** Menachem Sadeh, Ron Dabby

**Affiliations:** 1 Department of Neurology, Wolfson Medical Center, Holon 5822012, Israel; dabbyr@netvision.net.il; 2 Gray Faculty of Medical and Health Sciences, Tel Aviv University, Tel Aviv 6997801, Israel

**Keywords:** muscle biopsy, neuromuscular disorders, next-generation sequencing, inflammatory myopathies, immunohistochemistry, variants of uncertain significance (VUS)

## Abstract

This review examines the evolving role of muscle biopsy in the diagnosis of neuromuscular disorders in the era of modern genomic medicine. Historically the cornerstone of myopathy diagnosis, muscle biopsy enabled structural, histochemical, and ultrastructural characterization of muscle diseases. However, the introduction of next-generation sequencing and other genomic technologies has shifted the diagnostic paradigm, with genetic testing now serving as the preferred first-line approach for many hereditary myopathies due to its non-invasive nature and high diagnostic yield. However, muscle biopsy remains indispensable in the evaluation of inflammatory, toxic, metabolic, mitochondrial, and certain rare acquired myopathies. Biopsy is also valuable when genetic testing is inconclusive, particularly for interpreting variants of uncertain significance, through histopathological, immunohistochemical, and biochemical analyses. In certain disorders, diagnosis may rely primarily on biopsy findings. Emerging technologies, including RNA sequencing, transcriptomics, proteomics, spatial transcriptomics, and artificial intelligence-assisted pathology, are expanding the diagnostic value of muscle tissue beyond traditional morphological assessment. Rather than being replaced by genomic medicine, muscle biopsy is evolving into a complementary component of an integrated diagnostic strategy that combines clinical, pathological, and molecular data to improve diagnostic accuracy and guide precision medicine in neuromuscular disorders.

## 1. Introduction

The diagnostic landscape for neuromuscular disorders has undergone a profound transformation, moving from a reliance on macroscopic pathology to a sophisticated, multi-tiered approach that incorporates advanced molecular and genetic tools. For decades, the muscle biopsy procedure served as the unequivocal cornerstone of myopathology. Around seventy years ago, the development of techniques such as cryostat sectioning, histochemistry, and electron microscopy cemented the muscle biopsy as the primary method for describing and classifying a wide range of myopathies [1]. This historical foundation established a critical link between clinical symptomatology and observable, microscopic tissue pathology, a paradigm that dominated the field for generations.

The advent of next-generation sequencing (NGS) technology marked a new chapter, enabling the discovery of a rapidly increasing number of inherited myopathies [2]. This non-invasive diagnostic approach, often initiated with a simple blood sample, quickly became the preferred first-line test for many suspected hereditary disorders. For conditions like Duchenne muscular dystrophy (DMD), where the causative gene mutation is well-understood, genetic testing is now considered the “gold standard” [3]. This development has modified the traditional role of muscle biopsy, prompting a re-evaluation of its place in the diagnostic algorithm. Despite the efficiency and breadth of modern genetic testing, this review states that muscle biopsy has not been rendered obsolete. While genetic analysis now serves as the primary screening tool for many hereditary conditions, muscle biopsy remains indispensable for many conditions. This review delineates when and why each tool is most effective.

## 2. History of Muscle Biopsy and Contemporary Approaches

In the mid-19th century, the diagnosis of muscle disease relied entirely on clinical observation, with pathological confirmation available only at autopsy. This changed in 1865 when the French neurologist Guillaume Duchenne de Boulogne developed a specialized tissue punch (emporte-pièce histologique) to obtain muscle samples from living patients with what is now known as Duchenne muscular dystrophy (DMD). Microscopic examination revealed that the enlarged calf muscles were largely replaced by fat and connective tissue rather than hypertrophied muscle fibers, leading Duchenne to coin the term paralysie myoscléreuse [4,5]. Despite this pioneering achievement, muscle biopsy was not widely adopted because the procedure was invasive, painful, and associated with a high risk of infection in the pre-antiseptic era. Consequently, for many decades diagnosis continued to rely primarily on autopsy findings, with muscle biopsy used only in selected cases. By the late 19th and early 20th centuries, the development of reliable local anesthetics and antiseptic surgical techniques made open muscle biopsies far safer. The primary objective during this era was to answer whether the patient’s weakness was caused by a problem in the muscle itself or in the supplying nerves. Muscle biopsies were used to formalize the distinctions between neuropathic patterns such as “group atrophy” and myopathic patterns such as variation in fiber size, necrotic fibers, and increased connective tissue [6].

Throughout the first half of the 20th century, muscle biopsy remained firmly rooted in this structural paradigm. Tissues were fixed in formalin, embedded in paraffin wax, and stained with standard hematoxylin and eosin (H&E). The true golden age of myology began in the late 1950s and peaked in the 1970s. Two massive technical innovations collided: the widespread adoption of the cryostat (which allowed fresh muscle tissue to be snap-frozen in liquid nitrogen and sliced while frozen, preserving delicate cellular enzymes) and enzyme histochemistry. Biochemical stains were applied directly to frozen muscle slices. Instead of just looking at cell shapes, metabolic and enzymatic activity could be visualized [7,8]. This led to the landmark discovery that human skeletal muscle is composed of distinct fiber types: Type I (“slow-twitch”) fibers, rich in oxidative enzymes and specialized for endurance, and Type II (“fast-twitch”) fibers, enriched in glycolytic pathways, highly reactive for ATPase activity, and adapted for rapid, forceful contractions [1,8].

Equipped with stains like Gömöri trichrome, Periodic acid Schiff, Sudan black, Nicotinamide adenine dinucleotide dehydrogenase–tetrazolium reductase (NADH-TR), Cytochrome c oxidase (COX), succinate dehydrogenase (SDH), and Adenosine triphosphatases (ATPases), myologists began discovering entirely new categories of diseases that looked near normal under standard H&E stains. They identified several diseases that came to be named for their distinctive pathological features. “Central core disease,” described in 1956, was named after histochemical studies revealed central “cores” within muscle fibers that lacked oxidative enzyme activity [9,10]. The term “nemaline myopathy” was coined in 1963 after the modified Gomori trichrome stain demonstrated clusters of tiny thread-like red rods (“nemaline rods”) derived from Z-disk structural proteins [11]. Stains like Periodic acid–Schiff (PAS) and Oil Red O allowed clinicians to diagnose glycogen storage diseases (like Pompe or McArdle disease) and lipid storage myopathies by demonstrating abnormal accumulations of sugar or fat.

The integration of electron microscopy (EM) into the muscle biopsy workflow enabled visualization of the ultrastructural organization of the sarcomere and mitochondria. The combination of histochemical techniques and electron microscopy opened the field of mitochondrial medicine. Using the modified Gomori trichrome stain, the so-called “ragged-red fibers” were identified, leading to the emergence of the field of mitochondrial myopathies [12]. EM is still useful in selected cases to confirm the presence of abnormalities identified by light microscopy. Some myopathies can be diagnosed more readily and with greater accuracy using EM [13].

By the early 1980s, immunohistochemistry (IHC) and Western blotting had been introduced into muscle pathology. These techniques employed custom-engineered antibodies to determine whether specific structural proteins were present or absent in muscle tissue. In 1987, the missing protein in DMD was identified: dystrophin [14]. Muscle biopsies were subsequently stained with anti-dystrophin antibodies, and biopsies from boys with DMD showed a complete absence of sarcolemmal staining. Since then, IHC has been used for the diagnoses of muscular dystrophies through detection of absent or abnormally localized structural proteins (dystrophin, dysferlin, caveolin-3, and others) [15]. An immunohistochemical approach may enable clear discrimination between dystrophic myopathies associated with inflammation and primary inflammatory myopathies. In inflammatory myopathies, IHC distinguishes myositis subtypes through characteristic staining patterns, including expression of MHC classes I and II, C5b-9, and p62/TDP-43, as well as analysis of the inflammatory infiltrate [15].

### Ultrasound-Guided Muscle Biopsy and MRI-Directed Target Selection

In recent years, imaging-guided techniques have emerged to make muscle biopsies safer, less invasive, and more precise. Ultrasound (US)-guided percutaneous muscle biopsy is a minimally invasive technique that provides real-time visualization of the target muscle, needle trajectory, and adjacent neurovascular structures. It is usually performed as an outpatient or day-case procedure under local anesthesia, using a guillotine-type semiautomatic core needle (commonly 14 gauge) or a larger vacuum-assisted core system. Pre-procedural grayscale and Doppler US can identify the target muscle and help avoid vessels, nerves, and regions of fatty replacement or necrosis. Frequently sampled muscles include the vastus lateralis, tibialis anterior, gastrocnemius, and biceps brachii. When multiple cores are obtained, the tissue is generally sufficient for histological, immunohistochemical, and molecular analyses; reported histological adequacy in inflammatory myopathy series ranges from 74% to 100%, with good tolerability and no moderate or severe adverse events reported in the cited series [16,17].

Magnetic resonance imaging (MRI) is primarily used to plan, rather than to perform muscle biopsy. Its value is greatest when disease distribution is patchy or when distinguishing active pathology from chronic damage is essential. T2-weighted fat-saturated and short-tau inversion recovery sequences identify edema associated with active disease, whereas T1-weighted images demonstrate fatty infiltration and chronic structural change. MRI therefore facilitates selection of a muscle that is abnormal but not end-stage, often by targeting a moderately affected region with active signal change and limited fatty replacement. This approach can reduce, although not eliminate, sampling error and false-negative biopsy results [18].

MRI findings may be used to select the target muscle and approximate region, after which US is used for real-time needle guidance. In centers with MRI-US fusion capability, pre-procedural MRI can be co-registered with real-time US to target MRI-defined areas of active inflammation more precisely. This MRI-directed, US-guided strategy combines the superior tissue characterization and regional survey provided by MRI with the portability, lack of ionizing radiation, and real-time procedural control provided by US. It is particularly useful in inflammatory myopathies and muscular dystrophies with asymmetric or patchy involvement, as well as after a non-diagnostic prior biopsy [19].

US-guided biopsy is appropriate for suspected inflammatory, dystrophic, metabolic, or vasculitic myopathies when tissue diagnosis is expected to influence management. Its advantages include rapid access, avoidance of an operating room, the ability to sample a broad range of muscles, and suitability for repeat or serial assessment. Limitations include operator dependence, a smaller tissue yield than open biopsy, and the potential to miss focal disease without appropriate imaging-based selection. Open biopsy may remain preferable when a larger specimen or preservation of tissue architecture is critical. Contraindications include coagulopathy or uncontrolled anticoagulation, infection at the proposed biopsy site, inability to cooperate with the procedure, and complete fatty replacement of the selected muscle. MRI planning is also limited by cost, availability, contraindications, and the nonspecific nature of muscle edema [20].

## 3. History of Modern Genetics in the Diagnosis of Muscular Diseases

During the 1990s, polymerase chain reaction (PCR), Sanger sequencing, and Southern blot techniques enabled direct mutation detection in many hereditary myopathies, gradually reducing reliance on invasive muscle biopsy. The discovery of genes encoding sarcolemmal, nuclear, sarcomeric, and mitochondrial proteins revealed the marked genetic heterogeneity of muscular disorders and allowed for their classification according to molecular defects rather than solely pathological appearance. The introduction of Sanger sequencing enabled molecular diagnosis through single-gene testing. However, this approach was limited by its sequential nature: clinicians had to analyze genes one at a time based on clinical suspicion, phenotype, histological findings, and presumed inheritance patterns. With more than 170 genes associated with muscle disorders and substantial phenotypic overlaps between conditions, this strategy became increasingly impractical, time-consuming, and costly [21].

The development of NGS fundamentally transformed the diagnostic landscape of inherited neuromuscular diseases. Beginning around 2010, NGS enabled the simultaneous parallel sequencing of multiple genes through targeted gene panels. This technological advance substantially increased diagnostic yield, with gene panels achieving diagnostic rates of approximately 30–70%, while also reducing both cost and turnaround time compared with sequential single-gene testing [22].

Whole-exome sequencing (WES) was introduced shortly thereafter and became particularly valuable for unresolved or atypical neuromuscular cases. WES sequences the protein-coding regions of the genome (the exome), which comprise only 1–2% of the genome but harbor most known disease-causing variants. WES enabled the discovery of numerous novel neuromuscular disease genes and expanded the phenotypic spectrum of previously recognized disorders. It proved especially useful in congenital myopathies and complex pediatric neuromuscular syndromes in which the clinical phenotype did not clearly suggest a single candidate gene [23].

Whole-genome sequencing (WGS) represented the next major technological advance. Unlike WES, WGS sequences both coding and non-coding regions of the genome. WGS improved the detection of structural variants, repeat expansions, copy-number variations, deep intronic mutations, and mitochondrial DNA abnormalities that may be missed by exome sequencing. In neuromuscular disorders, WGS has become increasingly important for patients who remain genetically undiagnosed after conventional testing or WES. It offers comprehensive coverage of coding, non-coding, and intergenic regions, enabling improved detection of structural variants and simultaneous analysis of both nuclear and mitochondrial genomes [24].

More recently, the limitations of short-read sequencing platforms, particularly in detecting structural variants and repeat expansions, have driven the development of long-read sequencing technologies. These methods generate longer contiguous DNA reads and improve the detection of repetitive elements, copy-number alterations, and large expansions [25].

## 4. Genetic Testing and Muscle Biopsy: Diagnostic Approach

### 4.1. When to Start with Genetic Testing

Genetic testing is the preferred initial diagnostic approach when a hereditary neuromuscular disorder is suspected, and the phenotype is reasonably well characterized (e.g., “LGMD like”, congenital myopathy, dystrophinopathy, FSHD, OPMD, channelopathies) [26,27]. Key situations to begin with genetics:

Clear or pattern-based inherited phenotype (typical Duchenne/Becker, classic LGMD subtype, recognizable congenital myopathy pattern).

Family history suggesting Mendelian transmission.

Chronic course, and no strong red flags for acquired/inflammatory disease.

Availability of high-quality phenotype-driven NGS panels or exome sequencing and a strong expectation of monogenic or oligogenic etiology, such as in muscular dystrophies, many congenital myopathies, and channelopathies.

Situations where biopsy would not change the need for molecular confirmation (e.g., suspected dystrophinopathy in a boy: start with DMD deletion/duplication + sequencing; biopsy only if genetics is negative or discordant).

### 4.2. When to Start with Muscle Biopsy

Suspected inflammatory myopathy, especially when antibody panel results are delayed, inconclusive, discordant, seronegative, or only weakly positive.

Rapidly progressive weakness accompanied by markedly elevated creatine kinase (CK) levels, myopathic EMG findings, abnormal muscle MRI, systemic manifestations, or clinical red flags suggestive of vasculitis, immune-mediated necrotizing myopathy, overlap syndromes, or toxic myopathy, in which muscle biopsy findings may immediately guide immunotherapy decisions and alter clinical management [26].

Suspected metabolic or mitochondrial myopathy, particularly when histochemical and respiratory chain studies are required and mtDNA analysis or next-generation sequencing may need to be performed on muscle tissue rather than blood.

Although current guidelines often prioritize genetic testing before muscle biopsy, a pediatric cohort study found that genetic testing performed after biopsy had greater diagnostic utility because histopathological findings helped guide targeted molecular analysis. For example, ragged-red fibers prompted mitochondrial DNA testing, while multiminicores directed evaluation of congenital myopathy genes. In this setting, muscle biopsy provided critical clues that narrowed the differential diagnosis and enabled a more focused and efficient genetic workup [28].

It should be noted that certain dystrophies (dysferlinopathies, facioscapulohumeral muscular dystrophy) can show considerable inflammatory infiltrates on biopsy and even upregulation of major histocompatibility complex class I (MHC-I), complicating the distinction from inflammatory myopathies [29]. Biopsy of patients with calpainopathy due to homozygous pathogenic CAPN3 mutations may reveal infiltrates consisting of eosinophils [30] or macrophages and lymphocytes [31]. Accordingly, the presence of inflammatory infiltrates on biopsy does not exclude an underlying hereditary disorder [32].

### 4.3. Where Biopsy Adds Unique Value After Genetic Testing

Even in “genetics-first” diagnostic pathways, muscle biopsy often provides information that NGS cannot provide, or it helps interpret the genetic findings [33,34].

Dystrophinopathy with negative or inconclusive DMD testing: biopsy may be used to assess dystrophin expression and to support RNA-based detection of deep intronic or regulatory variants.

LGMD-like phenotypes with negative broad gene panels or exome sequencing: biopsy may reveal specific protein deficiencies (e.g., sarcoglycans, dysferlin, calpain 3, anoctamin-5), thereby guiding targeted re-analysis or additional genetic testing.

Variant interpretation: muscle biopsy may demonstrate protein absence, mislocalization, or characteristic structural changes that support the pathogenicity of a VUS or a candidate gene identified by exome/genome sequencing.

mRNA and cDNA analysis from muscle tissue can provide direct evidence of the functional effects of genetic variants, particularly in cases unresolved by DNA testing [35]. This approach detects splicing defects, exon skipping, deep intronic variants, and transcript loss due to nonsense-mediated decay that may be missed by standard sequencing. Because many neuromuscular genes are not expressed in blood, muscle tissue or myogenic cells derived from fibroblasts offer a more informative source and can improve diagnostic yield. RNA sequencing identifies pathogenic mechanisms in up to 38% of unresolved cases and can help reclassify variants of uncertain significance by demonstrating their functional impact. Emerging technologies, including transcriptomics, proteomics, and multi-omics approaches, are further expanding the diagnostic and research potential of muscle tissue analysis [33,35].

### 4.4. When Diagnosis Can Rest Essentially on Muscle Biopsy

There are clinical situations in which the diagnosis is established primarily by muscle biopsy, while genetic testing is either non-contributory or serves an adjunctive rather than decisive role.

Inflammatory myopathies: Polymyositis, dermatomyositis, immune-mediated necrotizing myopathy, or inclusion body myositis may be identified on biopsy, particularly when autoantibody panels are negative, delayed, or nonspecific. In such cases, treatment decisions can often be based on histopathological findings in conjunction with the clinical phenotype [32].

Classic dystrophinopathy with negative or inconclusive genetics: A marked reduction in or absence of dystrophin expression on immunohistochemistry in a boy with a Duchenne/Becker muscular dystrophy phenotype remains highly confirmatory for dystrophin deficiency. Subsequent genetic testing is then used mainly to define the precise variant for genetic counseling and mutation-specific therapies [36].

Metabolic and mitochondrial myopathies with demonstrable biochemical defects: Clear and reproducible respiratory chain deficiencies, ragged-red fibers, COX-negative fibers, or specific enzymatic defects identified in muscle tissue may establish a working diagnosis and guide management, even when the underlying genetic defect has not yet been identified [37].

Rare treatable entities with unresolved or negative genetics: Some disorders, such as lipid storage myopathy, may remain genetically unresolved despite extensive testing. In these cases, the diagnosis and consequently the initiation of targeted treatment may depend entirely on muscle biopsy findings [38].

Rare, acquired entities diagnosable only morphologically: Sporadic late-onset nemaline myopathy (SLONM), amyloid myopathy, and the recently described monoclonal gammopathy-associated glycogen storage myopathy (MGGSM) are potentially treatable disorders that may be clinically suspected but require muscle biopsy for definitive diagnosis [39].

This diagnostic approach is summarized in Figure 1.

## 5. Limitations of Muscle Biopsy

A significant proportion of muscle biopsies, up to approximately 20% in routine clinical practice, fail to yield a specific diagnosis, frequently returning “non-informative” or completely normal findings [40]. The limitations include:

*Sampling error due to patchy disease distribution*: Many myopathies exhibit highly focal or heterogeneous tissue involvement. A biopsy needle or incision can easily harvest entirely unaffected or non-specifically altered muscle tissue right alongside highly pathological lesions, leading to a false-negative result [41]. MRI may help reduce sampling error by identifying muscles with active disease and guiding optimal biopsy site selection. This is particularly valuable in inflammatory myopathies, where pathological changes are often patchy and unevenly distributed.

*Freezing artifacts*: Skeletal muscle tissue is highly sensitive to cryopreservation techniques. If a sample froze too slowly, the formation of ice crystals causes fine sarcoplasmic holes. This severe artifact can mask subtle structural features, such as metabolic defects or vacuolar changes, rendering the tissue uninterpretable [42].

*Small muscle sample*: Adequate muscle biopsy specimens should contain at least 200–250 well-oriented transverse muscle fibers. Fine-gauge needle biopsies often provide insufficient tissue, limiting optimal orientation and ancillary biochemical or mitochondrial studies, and increasing the risk of sampling error [43].

*Inappropriate site selection*: Selecting a muscle with severe, long-standing weakness can result in an “end-stage” biopsy where the primary pathological hallmarks have vanished, replaced entirely by non-specific fibrofatty tissue and marked atrophy.

### When to Consider a Repeat Muscle Biopsy

Repeat muscle biopsy is required in only a small proportion of myopathy evaluations and should be reserved for selected situations. Indications include a non-specific or non-diagnostic initial biopsy despite strong clinical suspicion of a treatable neuromuscular disorder, disease progression or unexpected treatment failure suggesting an incomplete diagnosis, and technically inadequate specimens affected by sampling or processing artifacts. Diagnostic yield may also improve when the repeat biopsy is guided by MRI or ultrasound to target active disease, or when an open surgical biopsy is performed after a non-diagnostic needle biopsy. In a large study, repeat biopsy was performed in 3.6% of cases and established a specific diagnosis in 43% [44].

## 6. The Role of Muscle Biopsy in Different Clinical Conditions

### 6.1. Muscle Biopsy in Rapidly Progressive Weakness

The main cause of rapidly progressive weakness, particularly when associated with markedly elevated CK levels and a myopathic EMG, is inflammatory or immune-mediated myopathy. Muscle biopsy remains essential for the diagnosis and subclassification of inflammatory myopathies. Each subtype of inflammatory myopathy demonstrates characteristic myopathological features that facilitate accurate classification. However, muscle biopsy is no longer mandatory for well-defined clinical-serologic presentations, such as classic dermatomyositis with characteristic rashes, or autoantibody-positive IMNM (anti-SRP or anti-HMGCR) with typical clinical features [32]. In patients with connective tissue disorders presenting with muscle weakness and elevated CK, biopsy is usually not performed. It nevertheless remains essential in patients without myositis-specific autoantibodies, in cases with delayed or inconclusive serologic results, and is particularly critical for the diagnosis of inclusion body myositis. Myositis-specific autoantibodies are found in only two-thirds of patients with myositis, making muscle biopsy particularly important in seronegative cases [45].

### 6.2. Dermatomyositis

Dermatomyositis is a disease mainly of skin and muscle but may affect lung and other tissues. The hallmark feature is perifascicular atrophy, characterized by layers of atrophic muscle fibers located at the periphery of fascicles. Inflammatory infiltrates are predominantly perivascular and interfascicular, in contrast to the endomysial pattern observed in other inflammatory myopathy subtypes. Vascular pathology is prominent and includes endothelial hyperplasia with tubuloreticular inclusions, fibrin thrombi, and capillary obliteration. MHC-I immunostaining demonstrates a characteristic perifascicular pattern, with increased expression in atrophic perifascicular fibers [32,45,46].

Recent studies have identified antibody-specific pathological variations. Anti-TIF1-γ dermatomyositis is characterized by vacuolated (“punched-out”) fibers and perifascicular MHC-I enhancement. Anti-Mi-2-associated disease shows prominent muscle damage, perifascicular necrosis, and increased perimysial alkaline phosphatase activity. Anti-MDA5 dermatomyositis demonstrates scattered or diffuse MxA staining with less severe muscle pathology, whereas anti-NXP-2 is associated with microinfarction [47].

### 6.3. Immune-Mediated Necrotizing Myopathy (IMNM)

IMNM is characterized primarily by widespread, simultaneous muscle fiber necrosis and active regeneration. Unlike other inflammatory myopathies, it features a striking lack of primary lymphocytic inflammation, with immune cells mostly limited to macrophages clearing necrotic debris (Figure 2). Diagnostic hallmarks include the deposition of the complement membrane attack complex (C5b-9) on the membranes of non-necrotic fibers and localized MHC-I expression. Immune-mediated necrotizing myopathy is associated with two specific antibodies: anti-SRP and anti-HMGCR [32,48]. It should be noted that, rarely, anti-HMGCR IMNM may present with a slowly progressive course and dystrophic biopsy features, thereby mimicking muscular dystrophy (Figure 3).

### 6.4. Anti-Synthetase Syndrome

Anti-synthetase syndrome is characterized by the presence of anti-aminoacyl-tRNA synthetase antibodies and a constellation of clinical features affecting multiple organ systems. The classic clinical presentation includes myositis, interstitial lung disease, arthritis, mechanic’s hands, Raynaud phenomenon, and fever.

Perifascicular necrosis and regeneration are the hallmark findings, present in about half of cases. Increased perimysial alkaline phosphatase activity is a distinctive feature, representing enhanced enzyme activity in the connective tissue surrounding fascicles.

Inflammatory infiltrates are variable in distribution and intensity. Some cases show myofiber necrosis similar to immune-mediated necrotizing myopathy. MHC-I upregulation can be demonstrated, though the pattern and extent vary. The pathological presentation is relatively homogeneous across different anti-synthetase antibodies [49].

### 6.5. Idiopathic Polymyositis

Idiopathic polymyositis refers to patients whose disease does not fit any of the other inflammatory myopathy subtypes and is therefore often considered a diagnosis of exclusion. This entity is now regarded as relatively rare [45]. Polymyositis shows endomysial inflammation with or without invasion of nonnecrotic fibers by mononuclear cells (usually CD8+ T cells) and with or without perimysial or perivascular inflammation invading nonnecrotic muscle fibers expressing MHC-I. Unlike dermatomyositis, inflammatory infiltrates are more within fascicles rather than perivascular/interfascicular. Necrotic fibers tend to be scattered or isolated rather than grouped [50].

### 6.6. Rhabdomyolysis

Advanced investigations are generally unnecessary when rhabdomyolysis is a single, isolated episode with a clear precipitating factor, such as major trauma, extreme exertion, toxin exposure, or an acute infection. In contrast, a comprehensive diagnostic evaluation is warranted when rhabdomyolysis is recurrent, occurs without a clear or proportionate trigger, fails to resolve completely, or is accompanied by baseline muscle symptoms or a family history of neuromuscular disease.

A useful clinical framework for identifying patients who require further investigation is the RHABDO mnemonic:

RHABDO criteria indicating the need for investigation of an underlying muscle disorder:

- R—Recurrent episodes of exertional rhabdomyolysis
- H—HyperCKemia persisting for more than 8 weeks after the event
- A—Accustomed to exercise (rhabdomyolysis occurring despite usual levels of physical activity)
- B—Blood CK concentration exceeding 50 times the upper limit of normal
- D—Drug ingestion insufficient to explain the exertional rhabdomyolysis
- O—Other family members affected or the presence of other exertional symptoms

Patients meeting one or more of these criteria should undergo further evaluation for an underlying genetic or metabolic myopathy.

Genetic testing is increasingly regarded as the preferred first-tier diagnostic approach in the evaluation of unexplained rhabdomyolysis. Targeted gene panels or NGS provide a non-invasive strategy with a high diagnostic yield, particularly for detecting muscular dystrophies, metabolic myopathies, and mitochondrial disorders. Muscle biopsy is generally reserved for patients with negative or inconclusive genetic findings.

Muscle biopsy enables “reverse phenotyping,” whereby histopathology, histochemistry, electron microscopy, and biochemical enzyme assays may identify a specific defect that guides more targeted genetic analysis or reveals structural and mitochondrial disorders that may not be adequately detected by standard gene panels. Correlation of tissue findings with genetic results can also help determine the pathogenicity of VUSs. In selected patients with suspected mitochondrial disease, particularly those with heteroplasmy, tissue-specific mtDNA mutation loads, or nuclear–mitochondrial interaction disorders, muscle tissue may still be required for respiratory chain enzymology, as these abnormalities may not be detected by blood-based genetic testing.

Muscle biopsy performed during or immediately after an acute episode of rhabdomyolysis is often uninformative or misleading because extensive myofiber necrosis and regeneration can obscure the underlying pathology. Consequently, biopsy is usually deferred for several weeks to months, until the patient has achieved clinical recovery [51,52].

### 6.7. Myalgia

Muscle biopsy has limited utility in isolated myalgia and should be reserved for selected cases with specific clinical, laboratory, or electrophysiological abnormalities. Myalgia is rarely caused by primary muscle disease; most cases arise from non-muscular sources, including ligaments, joints, bones, or peripheral nerves.

Muscle biopsy should be considered in patients with exertional myalgia when one or more of the following features are present: myoglobinuria, a second-wind phenomenon, objective muscle weakness, muscle hypertrophy or atrophy, elevated CK levels, or a myopathic EMG pattern.

The diagnostic yield of muscle biopsy in isolated myalgia without additional abnormalities is very low. In patients with chronic isolated myalgia and normal laboratory investigations, muscle biopsy findings were normal in 98–99% of cases [52,53].

### 6.8. Metabolic Myopathies

Rhabdomyolysis resulting from metabolic myopathies is discussed above. Mitochondrial myopathies can present with exercise intolerance and rhabdomyolysis. In these conditions, muscle biopsy may provide valuable diagnostic information. Gomori trichrome shows ragged red fibers in mitochondrial myopathies, PAS staining reveals glycogen accumulation in glycogen storage diseases, and increased lipid content on Oil-Red O or Sudan black suggests fatty acid oxidation disorders. In addition, muscle biopsy can play a pivotal role in the interpretation of VUS, providing functional and pathological evidence that may help establish pathogenicity [54].

### 6.9. Critical Illness Myopathy

Critical illness myopathy (CIM) is the most common non-inflammatory cause of rapidly progressive weakness in hospitalized patients, affecting 25–84% of patients requiring prolonged mechanical ventilation and up to 35% of those with status asthmaticus or COPD. CIM typically presents with generalized flaccid weakness and preserved sensation, when sensory examination is feasible. Risk factors include prolonged ICU stay, sepsis, multi-system organ dysfunction, hyperglycemia, and exposure to steroids and neuromuscular blocking agents. Muscle biopsy demonstrates myofiber necrosis and selective loss of myosin filaments [55].

### 6.10. Drug-Induced and Toxic Myopathies

Statin-induced myopathy can present acutely with rhabdomyolysis, particularly when combined with other medications (fibrates, azole antifungals, macrolides) or in the setting of hypothyroidism, renal impairment, or vitamin D deficiency. A subset of patients with statin myopathy had histopathologic or electron microscopic evidence for mitochondrial dysfunction in skeletal muscle [56].

Other toxic myopathies are associated with alcohol, colchicine, antimalarial agents (chloroquine and hydroxychloroquine), and certain chemotherapeutic drugs, all of which may cause acute or subacute muscle weakness. In most cases, clinical improvement follows withdrawal of the offending agent. However, if weakness fails to improve within several weeks after drug discontinuation or continues to progress despite cessation of therapy, muscle biopsy should be considered to exclude an alternative or coexisting myopathic process.

The hallmark pathological feature of colchicine-induced myopathy is a vacuolar myopathy characterized by the accumulation of autophagic vacuoles in the absence of significant muscle fiber necrosis. These vacuoles predominantly involve type 1 muscle fibers and stain strongly for acid phosphatase, reflecting their lysosomal origin. Similar autophagic vacuolar changes may also be observed in chloroquine- and hydroxychloroquine-induced myopathies [57,58]. LC3 and p62 are established markers of autophagosome accumulation. Immunohistochemical staining for these proteins demonstrates characteristic punctate sarcoplasmic aggregates, providing evidence of impaired autophagic flux and autophagosome accumulation without the need for electron microscopy [59].

### 6.11. Endocrine Myopathies

Endocrine myopathies can closely mimic inflammatory myopathies and may lead to misdiagnosis if thyroid and metabolic function tests are not obtained early in the diagnostic evaluation. Patients with severe hypothyroidism may present with proximal muscle weakness, markedly elevated CK levels, and a necrotizing pattern on muscle biopsy [60]. Consequently, they may be incorrectly diagnosed with IMNM if thyroid dysfunction is not recognized. Importantly, central hypothyroidism can also be associated with necrotizing myopathy despite a normal thyroid-stimulating hormone (TSH) level, underscoring the need to assess free thyroid hormone levels when clinical suspicion persists [61].

Hyperthyroidism may likewise cause profound, rapidly progressive weakness, occasionally manifesting as thyrotoxic periodic paralysis, which is often precipitated by carbohydrate-rich meals or rest following exercise. The pathological findings in hyperthyroid myopathy are nonspecific and variable, ranging from minimal myopathic changes to more pronounced structural abnormalities. Unlike drug-induced vacuolar myopathies, hyperthyroid myopathy lacks characteristic histopathological features, and muscle biopsy is rarely required for diagnosis because the clinical presentation and laboratory findings are usually sufficient to establish the underlying endocrine disorder [62].

### 6.12. Hereditary Myopathies with Rapidly Progressive Course

Most hereditary myopathies follow a slowly progressive clinical course. However, a subset may present with relatively rapid disease progression. This poses a significant diagnostic challenge, as distinguishing hereditary myopathies from seronegative inflammatory myopathies is crucial for establishing an accurate diagnosis and for initiating appropriate targeted therapy when indicated. Muscle biopsy remains a valuable tool in making this distinction.

An example of a hereditary disease that may progress rapidly is reducing body myopathy (RBM). This is an X-linked dominant muscle disorder caused by a pathogenic variant in the *FHL1* gene. Pathologically, it is defined by reducing bodies: intracytoplasmic inclusions in muscle fibers that reduce nitro-blue tetrazolium (NBT) and stain intensely with menadione–NBT. The clinical spectrum varies widely, from mild, asymmetric proximal weakness to severe, progressive myopathy with respiratory failure and cardiac involvement. Presentation in women can be influenced by skewed X-inactivation. A muscle biopsy is essential for promptly differentiating RBM from inflammatory or other hereditary myopathies [63,64] (Figure 4).

## 7. Muscle Biopsy in Slowly Progressive Myopathies

### 7.1. Muscular Dystrophies

Muscular dystrophies are a heterogeneous group of inherited muscle disorders characterized by progressive muscle weakness and degeneration. In most cases, muscle biopsy demonstrates a dystrophic pattern; however, these histopathological changes are often nonspecific and insufficient to establish a precise diagnosis.

Immunohistochemistry and immunoblotting can provide valuable diagnostic information by identifying deficiencies or abnormalities of specific muscle proteins. These techniques are particularly useful in autosomal recessive muscular dystrophies, where the causative protein defect is known and reliable antibodies are available for detection.

With the widespread availability of next-generation sequencing, muscle biopsy has shifted from a primary diagnostic tool to a complementary investigation, whereas genetic testing has become the first-line diagnostic approach for most muscular dystrophies. In disorders with characteristic clinical phenotypes, such as myotonic dystrophy, facioscapulohumeral muscular dystrophy (FSHD), and oculopharyngeal muscular dystrophy (OPMD), genetic testing should be performed as the initial diagnostic investigation, without a preceding muscle biopsy [26].

#### 7.1.1. Negative or Inconclusive Genetic Testing

Current indications for muscle biopsy in muscular dystrophies include:

When clinical suspicion for a muscular dystrophy remains high despite nondiagnostic genetic results, muscle biopsy may reveal protein abnormalities that can guide further genetic investigations.

#### 7.1.2. Suspected Dystrophinopathy with Negative Genetic Testing

In patients with a clinical phenotype suggestive of Duchenne muscular dystrophy (DMD) or Becker muscular dystrophy, muscle biopsy with dystrophin immunohistochemistry and Western blot analysis is indicated when routine genetic testing is unrevealing (Figure 5). Rare deep intronic variants or complex structural rearrangements may escape detection by standard genetic methods [65]. Moreover, distinguishing complete from partial dystrophin deficiency can help differentiate DMD from milder dystrophinopathy phenotypes.

#### 7.1.3. Interpretation of VUS

Muscle biopsy may demonstrate specific protein abnormalities or structural changes that support the pathogenicity of variants identified by next-generation sequencing. Immunohistochemical and immunoblot analyses provide direct evidence of protein expression and integrity that cannot be inferred from DNA sequencing alone [66].

An integrated diagnostic approach that combines clinical phenotype, age at onset, pattern of muscle weakness, serum CK levels, muscle imaging findings, genetic testing, and the selective use of muscle biopsy remains the optimal strategy for the evaluation of muscular dystrophies.

### 7.2. Congenital Myopathies

The role of muscle biopsy has shifted from the primary gold standard for diagnosing congenital myopathies to an integrative tool within the modern diagnostic landscape. While advanced genetic testing has fundamentally changed the diagnostic workflow, muscle biopsy remains indispensable for managing the genetic and clinical heterogeneity characteristic of these disorders [34].

Muscle biopsy is critical for resolving the pathogenicity of VUSs identified during genetic testing. Histological and ultrastructural analysis can confirm if a genetic variant correlates with observed structural protein dysfunction [67]. In cases where clinical presentation is ambiguous, morphological and histochemical findings (e.g., nemaline bodies, central cores, or fiber-type disproportion) can help prioritize candidate gene panels, and facilitate interpretation of genetic findings [34]. It remains vital for distinguishing congenital myopathies from other neuromuscular conditions such as inflammatory myopathies, metabolic disorders, or neuropathic processes when genetic results are inconclusive or absent [67].

### 7.3. Myofibrillar Myopathies

In the pre-genomic era, muscle biopsy was regarded as the diagnostic gold standard for myofibrillar myopathies (MFMs) [68]. Diagnosis relied on the identification of characteristic pathological features by light and electron microscopy, including focal myofibrillar dissolution originating at the Z-disk, progressive disorganization of the myofibrillar network, and abnormal intracellular accumulation of intermediate filaments and chaperone proteins such as desmin, αB-crystallin, and myotilin [69]. However, MFMs are clinically, genetically, and pathologically heterogeneous. No specific histopathological pattern reliably predicts the underlying genetic defect or clinical phenotype, and pathogenic findings in classical MFM-associated genes may occur without typical myofibrillar pathology.

The introduction of targeted gene panels, WES, and WGS has transformed the diagnosis of myofibrillar myopathies, although many cases remain genetically unresolved [69,70]. In these patients, muscle biopsy remains crucial for confirming MFM, guiding genetic testing, and aiding interpretation of VUS.

### 7.4. GNE Myopathy

Genetic testing is now considered the main diagnostic approach representing a shift from the pre-genetic era when muscle biopsy was essential for the diagnosis. Nevertheless, muscle biopsy retains an important role when VUSs are identified on molecular genetic testing. The characteristic histopathologic findings including rimmed vacuoles on modified Gomori trichrome staining, fiber size variation, atrophy, and absence of inflammation, can provide supportive evidence for variant pathogenicity. However, a biopsy performed too early or on an inappropriate muscle (e.g., the heavily spared quadriceps femoris) can lead to false-negative histopathology (e.g., absence of vacuoles). As awareness of GNE myopathy increases among clinicians, the role of muscle biopsy will likely continue to diminish, reserved primarily for cases with uncertain genetic findings [71,72].

### 7.5. Lipid Storage Myopathies

Multiple acyl-coenzyme A dehydrogenase deficiency (MADD) is a rare autosomal recessive fatty acid oxidation disorder, caused by defects in electron transfer flavoprotein (ETF) genes. As a result, electron transfer from multiple flavoprotein dehydrogenases to the mitochondrial respiratory chain is impaired, leading to defective oxidation of fatty acids and other metabolic substrates. It presents in severe neonatal forms with or without congenital anomalies or as late-onset forms. A subset of patients may present with a pure myopathic form with progressive proximal weakness which may be associated with axonal sensory neuropathy.

Genetic testing may identify pathogenic variants in the ETFDH, ETFA, or ETFB genes. However, several reports have described patients with clinically and biochemically confirmed MADD who carry only a single heterozygous variant or in whom no pathogenic variants are detected. In such cases, the diagnosis has been established by muscle biopsy demonstrating lipid accumulation within muscle fibers, together with supportive findings on acylcarnitine profiling and a favorable response to therapy. Prompt muscle biopsy is particularly important in these patients, as it can facilitate the diagnosis of a potentially treatable disorder and enable the initiation of highly effective therapy (Figure 6). Recent findings indicate that sertraline (an SSRI antidepressant) can trigger this myopathy [38,73].

### 7.6. Inclusion-Body Myositis

Inclusion-body myositis (IBM) is a slowly progressive, asymmetric myopathy that typically affects the quadriceps and finger flexors. Although its etiology remains unclear, genetic susceptibility has been linked to HLA-DRB1*03:01, which is associated with earlier onset and more severe disease [74]. Pathologically, IBM combines inflammatory and degenerative features that distinguish it from other inflammatory myopathies. The inflammatory component consists of endomysial CD8+ T-cell infiltrates invading MHC-I-expressing non-necrotic fibers, often with additional perivascular inflammation. The degenerative component includes rimmed vacuoles, endomysial fibrosis, fiber size variability, and atrophic angulated fibers, although rimmed vacuoles are absent in up to half of cases. Mitochondrial abnormalities such as ragged-red and COX-negative fibers are common, and electron microscopy may show 15–18 nm tubulofilamentous inclusions. Protein aggregates containing p62, TDP-43, and amyloid deposits further support the mixed inflammatory–degenerative nature of the disease [75,76] (Figure 7). Anti-cN1A antibodies are highly specific (92–98%) but only moderately sensitive (30–70%) for IBM, making them useful for confirming the diagnosis when positive but insufficient to rule it out when negative [32].

### 7.7. Sporadic Late-Onset Nemaline Myopathy

Sporadic late-onset nemaline myopathy (SLONM) is a rare, acquired, adult-onset muscle disease characterized by subacute progressive proximal and axial weakness, with nearly half of cases associated with monoclonal gammopathy. The disease manifests with prominent neck extensor and paraspinal muscle involvement, often causing head drop or camptocormia, along with dysphagia, dyspnea, and respiratory compromise in many patients. Muscle biopsy is essential and diagnostic, demonstrating the pathologic hallmark of nemaline rods (aggregates of degenerated Z-disk and thin filament proteins) in atrophic muscle fibers. However, diagnosis can be challenging because rod-containing fibers may be sparse and patchily distributed, with a considerable number of patients requiring EM or repeat muscle biopsies to establish the diagnosis. Immunohistochemical staining for α-actinin significantly enhances diagnostic sensitivity when rods are not readily apparent on standard modified Gömöri trichrome staining. Importantly, SLONM is a treatable condition that responds to immunomodulatory therapies including intravenous immunoglobulins, chemotherapy, or autologous stem cell transplantation, making timely biopsy-confirmed diagnosis critical to prevent diagnostic delay and initiate appropriate treatment for this otherwise potentially lethal disease [77,78].

### 7.8. Amyloidosis

Amyloidosis encompasses a diverse group of disorders caused by abnormal protein folding and aggregation. Histopathologically, these conditions are defined by the extracellular accumulation of amyloid fibrils, which form Congo red-positive amorphous deposits within various organs and tissues. These deposits can impair organ function and, in systemic forms, may result in significant morbidity and mortality. The peripheral nervous system is frequently affected in systemic amyloidosis, while involvement of skeletal muscle is uncommon. Among neuromuscular manifestations, immunoglobulin light-chain (AL) amyloidosis and hereditary transthyretin amyloidosis (ATTRv) represent the most prevalent systemic subtypes. In contrast, amyloid deposition may occasionally be confined to skeletal muscle, a condition termed isolated amyloid myopathy.

AL amyloidosis myopathy typically causes gradual or subacute proximal muscle weakness and dysphagia, and may involve facial, neck, or tongue muscles. Rarely can it present as rapidly progressive proximal weakness with elevated CK [79]. Peripheral neuropathy occurs in about one-third of patients. This condition should be suspected in patients over 40 presenting with unexplained myopathy, especially alongside neuropathy, cardiomyopathy, dysphagia, or weight loss [80]. Diagnosis requires a muscle biopsy, though Congo red staining is not always routinely done. The biopsy serves both to identify amyloid deposits and to characterize the amyloid subtype.

Transthyretin amyloidosis is characterized by the deposition of misfolded transthyretin (TTR) proteins in tissues, resulting in progressive organ dysfunction. ATTR amyloidosis occurs in two forms: hereditary (ATTRv) and acquired wild-type (ATTRwt) amyloidosis. ATTRv amyloidosis is caused by pathogenic variants in the TTR gene and follows an autosomal dominant inheritance pattern with variable penetrance. ATTRwt amyloidosis, formerly referred to as senile cardiac amyloidosis, is associated with age-related destabilization of the transthyretin tetramer, which promotes misfolding and amyloid fibril formation.

Precise identification of the amyloid subtype in muscle and nerve biopsy specimens is critical for selecting the appropriate therapy, as management differs markedly among amyloid disorders. Prompt diagnosis and early initiation of treatment are vital to prevent irreversible organ damage and improve survival. Liquid chromatography–tandem mass spectrometry (LC- MS/MS) and immunohistochemistry are currently used for amyloid subtyping. Both methods require paraffin-embedded tissue [81].

In conclusion, amyloid myopathy, although rare, should be considered in patients with otherwise unexplained muscle weakness, particularly when accompanied by systemic features. Early recognition and muscle biopsy with Congo red staining are crucial, as amyloid deposits may be overlooked without targeted evaluation [82].

### 7.9. Monoclonal Gammopathy-Associated Glycogen Storage Myopathy

Monoclonal gammopathy-associated glycogen storage myopathy (MGGSM) is a recently recognized, treatable acquired myopathy linked to monoclonal gammopathy, first described in 2023 [83]. It presents in adults with subacute proximal and axial weakness, often accompanied by weight loss, muscle stiffness, dysphagia, or head drop. Muscle biopsy is diagnostic, showing PAS-positive glycogen-filled vacuoles, autophagic material, and granular sarcolemmal MAC staining, while genetic testing for inherited glycogen storage diseases is negative. The condition often responds well to immunotherapy or autologous stem cell transplantation [84,85].

## 8. Future Perspectives

Because genetic testing often identifies variants of uncertain significance (VUSs) or does not fully explain the clinical phenotype, advanced analysis of muscle tissue has become increasingly important. Muscle biopsy is evolving from a purely structural examination into a powerful molecular tool. For example, RNA sequencing can provide a diagnosis in 35–40% of cases that remain unsolved after exome sequencing by detecting abnormal splicing, deep intronic variants, and other transcript-level defects not visible in DNA analysis [35]. RNA sequencing of muscle tissue identified a recurrent deep intronic defect in COL6A1 that caused inclusion of a harmful pseudoexon, leading to the diagnosis of previously unexplained cases of COL6-related muscular dystrophy [33].

New technologies such as spatial transcriptomics and proteomics preserve tissue architecture while revealing molecular changes within their anatomical context. Combining genomic, transcriptomic, proteomic, and metabolomic data provides a comprehensive view of disease, linking genetic variants to downstream effects on proteins, cellular pathways, and metabolism [86,87].

Artificial intelligence (AI) is also transforming muscle pathology. Deep-learning algorithms can analyze digital biopsy slides, quantify pathological features, map inflammatory changes, and even predict underlying molecular signatures. As a result, muscle biopsy is becoming not only a tool for detecting structural abnormalities but also a functional platform that helps interpret genetic findings and guide precision therapies [88].

Traditional histopathology remains essential for evaluating disease pathology. The future of muscle biopsy lies in integrating advanced molecular technologies with morphological assessment to support precision medicine in neuromuscular disorders.

## 9. Conclusions

The diagnostic approach to myopathies has undergone a profound transformation with the advent of next-generation sequencing and other genomic technologies. Although genetic testing has become the preferred first-line investigation in many inherited muscle disorders, muscle biopsy continues to play a pivotal role in selected clinical settings. Beyond its established value in inflammatory, toxic, metabolic, and mitochondrial myopathies, muscle biopsy remains an important tool for interpreting variants of uncertain significance, guiding targeted molecular investigations, and providing functional evidence of disease mechanisms. Emerging technologies, including transcriptomics, proteomics, and multi-omics approaches, are further expanding the diagnostic and research potential of muscle tissue analysis. Rather than being replaced by genomic medicine, muscle biopsy is evolving into a complementary and increasingly sophisticated component of an integrated diagnostic strategy. The future of neuromuscular diagnosis will likely depend not on choosing between biopsy and genetics but on combining clinical, pathological, and molecular data to achieve the highest diagnostic accuracy and improve patient care.

## Figures and Tables

**Figure 1:**
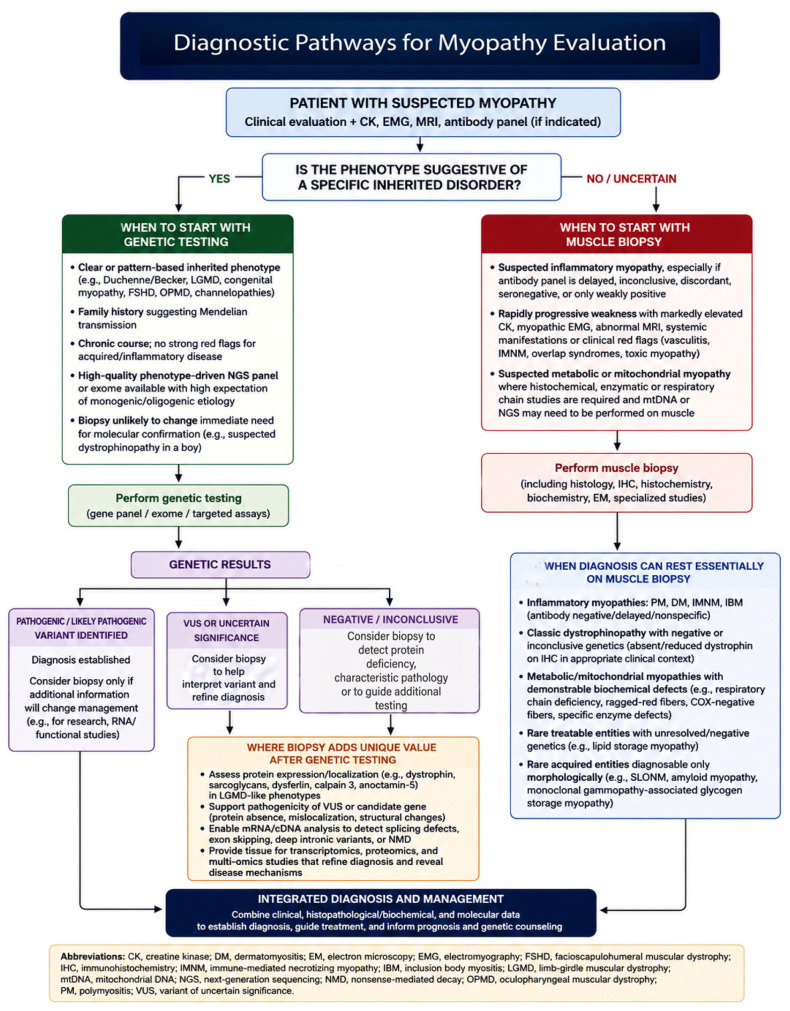
Diagnostic algorithm for the evaluation of myopathy.

**Figure 2:**
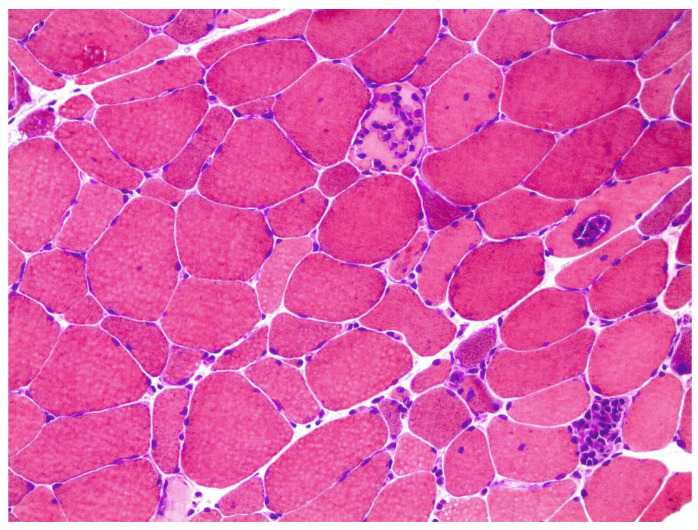
Immune-mediated necrotizing myopathy. Necrotic muscle fibers are infiltrated by macrophages. H&E.

**Figure 3:**
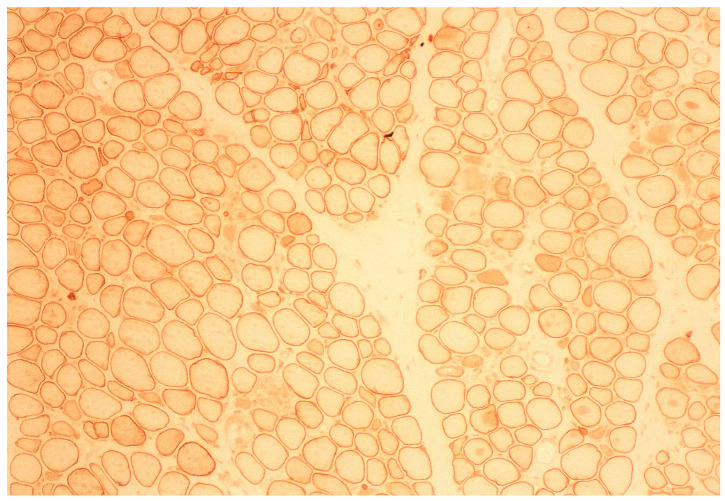
Dystrophin immunostaining in anti-HMGCR IMNM demonstrating dystrophy-like changes, including marked fiber size variation, rounded fibers, increased fibrous tissue, and scattered necrotic fibers.

**Figure 4:**
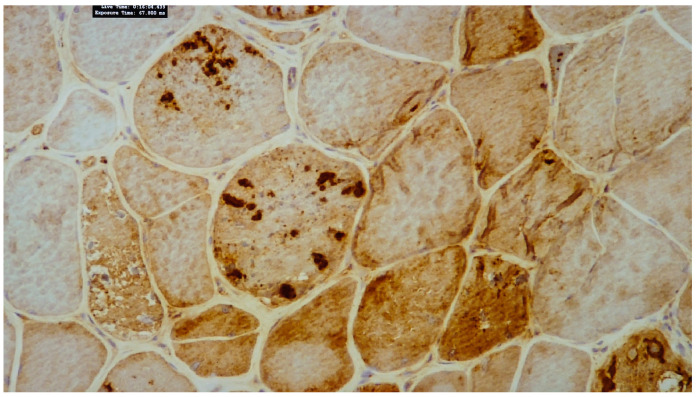
Reducing body myopathy. Myotilin immunostaining highlights irregularly shaped cytoplasmic inclusions within muscle fibers of variable sizes.

**Figure 5:**
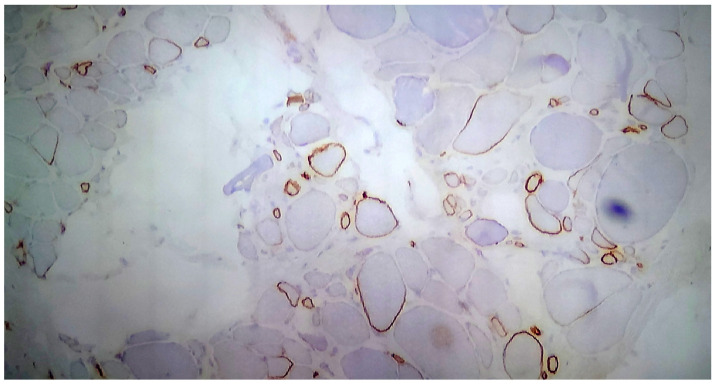
Dystrophin-2 immunostaining of a muscle biopsy from a patient with Becker muscular dystrophy demonstrating reduced and patchy sarcolemmal expression of dystrophin.

**Figure 6:**
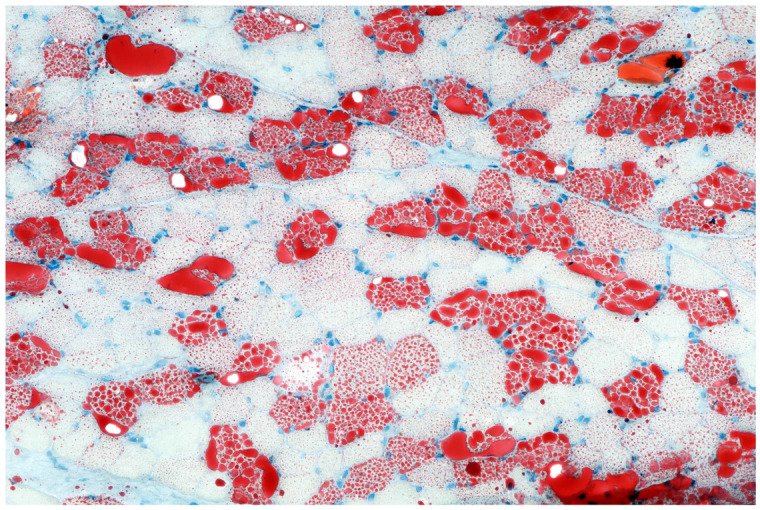
Oil Red O stain of a muscle biopsy showing numerous small and large lipid-filled vacuoles predominantly within type 1 muscle fibers.

**Figure 7:**
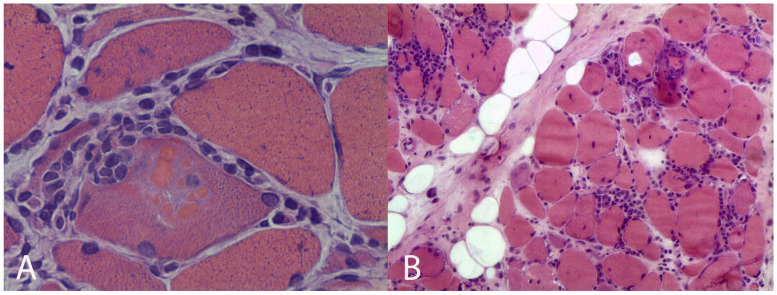
Inclusion body myositis (IBM). (**A**) Endomysial inflammatory infiltrates surrounding non-necrotic muscle fibers, with focal invasion of individual fibers. (**B**) Cytoplasmic aggregates within muscle fibers in the absence of rimmed vacuoles. H&E.

## Data Availability

No new data were created or analyzed in this study. Data sharing is not applicable to this article.
